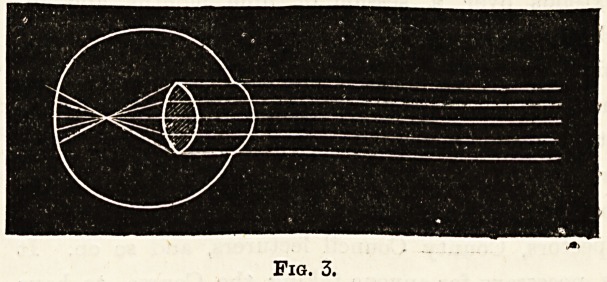# Nursing Section

**Published:** 1904-03-05

**Authors:** 


### The Hospital.

### Nursing Section.

Contributions for this Section of "The Hospital" should be addressed to the Editor, "The Hospital" Nursing Section, 28 & 29 Southampton Street, Strand, London, W.C.

No. 910.—Vol. XXXV. *SATURDAY, MARCH* 5, 1904.

### Notes on News from the Nursing World.

### PRINCESS CHRISTIAN'S NURSES.

The new medical and surgical home erected by Princess Christian, in connection with the district nurses' establishment in Windsor, as a memorial of Prince Christian Victor, was opened by her Royal Highness on Saturday last. After a short religious service, an illuminated address was presented to the Princess, congratulating her upon the completion of the work which has for so long a time occupied her thoughts, and warmly acknowledging the benefit conferred upon the town and neighbourhood of Windsor by the district nurses. Her Royal Highness having formally declared the home open presented long-service medals to four of the nurses—Miss Gleave, Miss Duckett, Miss Carpenter, and Miss Douglas—subsequently conducting the company through the home, which includes an operating-room. Over the chief entrance to the block of buildings is the monogram "H. C.," surmounted by a crown, and within is a brass tablet engraved with the inscription: "To the ever dear memory of her beloved son, Christian Victor, this nursing home has been founded by his mother, Helena, Princess Christian of Great Britain and Ireland, Feb. 27th, 1904."

### THREATENED WITHDRAWAL OF NURSES IN EAST LONDON.

The annual meeting of the East London Nursing Society will be held at the Mansion House on Tuesday afternoon, March 15th, the Lord Mayor in the chair. Among the speakers will be the Archdeacon of London, Dr. Robert Hutchison, and, it is hoped, General the Hon. Sir Reginald Talbot, the new Governor of Victoria, who had lately accepted the office of treasurer to the society, but has now necessarily resigned it. The number of cases nursed last year was 4,652, or 549 more than in the previous year, while the number of visits paid exceeded those of 1903 by 11,400. There are at present 27 nurses working in 34 parishes, but we regret to learn that the state of the finances of the association is a matter of grave anxiety. The expenditure exceeded the income to the extent of £332 during the twelve months, and the deficit has had to be met by accumulations from the "Montrose" Fund, which was not intended for that purpose. In these circumstances, unless there is an immediate increase of income of at least £350 per annum, some of the nurses will have to be withdrawn. We agree with the committee that this would be "a disastrous step," and we hope that the support given at the annual meeting will be liberal enough to avert it. Already a nurse doing good work in the outlying district of Stratford has been withdrawn partly owing to want of funds, but in view of the increasing needs it should be possible rather to augment than to diminish the supply of nurses.

### THE MATRON'S SIGNATURE ON CERTIFICATES.

For a considerable period the Croydon Guardians have given their probationers certificates not having the matron's signature. From time to time the subject has been raised, and quite recently, as our readers know, a probationer wrote to the Guardians, after completing her period of training, requesting that her certificate should be signed by the matron. The Board decided to make no change. It was, however, pointed out in The Hospital that issuing certificates without the matron's signature was doing an injustice to the Croydon infirmary as a training school, as well as to the probationers by handicapping them. We understand that the matter has since been brought to the notice of the Guardians by several probationers who have left as they find a difficulty in getting engagements because of the character of the certificate they possess; and that more lately the probationers now at the Croydon Infirmary forwarded a petition asking for certificates to be signed in future by the matron. This petition came before the Infirmary Committee, and on Tuesday they recommended to the Guardians that steps should be taken to comply with the prayer of it. The Guardians considered the matter in camera, and decided, but only by the casting vote of the Chairman, not to approve of the recommendation for the present. This should encourage the nursing staff to persevere.

### MAKING IT HARD FOR JUNIOR NURSES.

The latest development of the "ragging" case at Tooting Bec Asylum for Imbeciles is that the junior nurse who was the subject of the outrage, respecting which we gave the official statement last week, has left the institution. It appears that she failed to report herself at the time when she returned on the occasion of her leave, and gained admission to the nurses' quarters through the window instead of by the door. This breach of discipline had the not surprising effect of expediting her departure. Although recent events do not convey a favourable impression of the tone of the nursing staff at Tooting Bec Asylum, we believe that this is the first trouble. Also it may at least be pleaded on their behalf that they have had neither the training, nor the education, of hospital nurses. In the not far distant future we hope that all asylum attendants—even those employed to look after aged imbeciles—will enjoy the advantages of adequate training, and we hope that the gradual accession to their ranks of trained and educated nurses will make both for greater efficiency and better discipline. It is not, however, encouraging to know that the practice of hectoring junior nurses extends to hospitals which are recognised as schools of the highest class. There has just come under our notice the case of a probationer who, strongly imbued with a love of nursing, from the highest motives, and a keen sense of duty, has had to leave the hospital where she was on trial for training, not because the matron was dissatisfied with her, but because she was alleged not to have treated her "superiors" in the ward with becoming respect. The basis of this accusation was that, in ignorance, she had filled a hot-water bottle, and cut a patient's nails, when requested, without first having asked permission of her senior. Junior nurses suffer from inexperience, if not from flightiness, but training corrects inexperience, and discipline can be maintained without hectoring on the part of those who, if only because of better knowledge, should be patient and considerate to the beginners under them.

### THE NURSES OF THE EVELINA HOSPITAL.

The new quarters erected for the nursing staff of the Evelina Hospital for Children were open to inspection on Wednesday afternoon last week, when a large number of visitors availed themselves of the opportunity of going over the hospital. The occasion was the renaming of one of the wards, in memory of Mrs. Zunz, £5,000 of the Zunz Bequest having been awarded by the trustees to this hospital. The nurses' quarters are on the top floor of the building, and are both light and airy. There are twenty-four bedrooms, a large sitting-room, three bathrooms, and other offices. There is a separate bathroom for the night nurses, whose rooms are shut off by glass doors. All the bedrooms are coloured buff, the floors being covered with linoleum. There is a rail for pictures, and the furniture is simple but convenient. A red colour wash has been chosen for the walls of the sitting-room, which is amply provided with easy chairs. A cage of canaries gives a cheerful finish to this particularly cosy apartment. Among the many visitors on Wednesday were Miss Monk, matron of King's College Hospital, Miss Rowe, matron of the East London Hospital for Children, Miss L. Halliday, matron of the Royal Waterloo Hospital, and Miss Cross, for 24 years lady superintendent of the Evelina Hospital, while the opening ceremony was performed by Mrs. Leopold de Rothschild. Tea and coffee were dispensed by the sister-matron, Miss Getz, and the nurses.

### THE PROPOSED AMENDMENT OF THE MIDWIVES ACT.

At the meeting of the Central Midwives Board, last week, the question of the payment of a registered medical practitioner sent for on the advice of a midwife in compliance with Section E, Rule 17, of the rules of the Board was considered, and after discussion it was resolved unanimously "that inasmuch as no provision is made in the Midwives Act, 1902, for the payment of legally qualified medical practitioners, the Government be requested to take the necessary steps for amending the Act, by introducing a section or otherwise, to provide for the payment of legally qualified medical practitioners when called in by midwives in difficult cases." The Secretary was directed to send a copy of the foregoing resolution to the Privy Council. Before this important resolution was passed the Board discussed a letter from the President and the Secretary of the Royal Academy of Medicine in Ireland reciting the difficulties that will be experienced by the Irish Chartered Maternity Hospitals in enabling their pupil midwives to comply with the rules of the Board as to personal delivery of 20 cases and a 10 days' puerperium. But in the result the Board merely expressed again regret that the suggested alterations in the rules were not brought to their notice before the rules were sent to the Privy Council, and reiterated the opinion that it was now impossible for the Board to alter them. If, however, Parliament is to be asked to amend the Act in one direction, it can in another. The Board have ordered for entry on the roll the names of 917 more women. Of this total 1 claimed as holding the certificate of the Royal College of Physicians of Ireland, 291 that of the London Obstetrical Society, 9 that of the Rotunda Hospital, Dublin, 4 that of the Coombe Hospital, Dublin, 14 that of Queen Charlotte's Hospital, 9 that of the Liverpool Lying-in Hospital, 2 that of the British Lying-in Hospital, 3 that of the Glasgow Maternity Hospital, 11 that of St. Mary's Hospital, Manchester, 3 that of the City of London Hospital, 1 that of the Salvation Army Hospital, and 569 were admitted as having been in *bonâ fide* practice for one year prior to July 31, 1902.

### NURSES ADVISED TO ASK FOR CREDIT.

A curious discussion has taken place at a meeting of the Atcham Guardians in reference to the payment of salaries to the nurses. It seems that five of the eight nurses had written to ask the Guardians if their salaries might be paid monthly instead of quarterly. No objection to the little additional labour involved was offered by the Clerk to the Guardians, who pointed out that as their cheques did not require to be stamped, monthly payments would not entail an enlarged expenditure in penny stamps. In spite of this, the chairman contended that the Board "could not afford the extra expense the change would entail," while another member said that the proposed change would not "mean much to the nurses." The chairman, endorsing this, also suggested that "somebody will give the nurses a little more credit if they want it," and the request was not acceded to. We think that it might have been complied with, and we are surprised that the chairman should have talked so lightly about the nurses getting into debt. It is not only that, like other people, they have to pay for credit; but the principle of a nurse asking for credit is bad, and certainly ought not to receive encouragement from the chairman of a Board of Guardians.

### RETROGRESSION IN WORKHOUSE NURSING.

In the nursing returns for 1904 compiled from returns supplied by the clerks to the Guardians, Mr. H. Jenner-Fust, Local Government Board Inspector for the district comprising the union counties of Lancaster and Westmorland, together with part of Cumberland, states that the increase in the number of nurses has again "just kept pace with the increased number of sick." But he also reports that there is still great need in several instances for more night nurses, and he notes, with regret, an increase in the number of paupers employed under the name of attendants. We observe from his figures that in January 1902 the number of these attendants had diminished from 191 to 170, and that in 1903 there had been a further decrease to 152. In January 1904 the number was 175. This is retrogression, and indicates that some of the Boards of Guardians in Mr. Jenner-Fust's district would like to relapse into the old bad ways if they could.

### AUCKLAND WORKING MEN'S NURSING ASSOCIATION.

The working men of Bishop Auckland must be congratulated upon the figures of their first annual report, and the proceedings at the first annual meeting of the Auckland and District Working Men's Nursing Association. The report shows that 325 cases were dealt with by the nurses, and that the total number of visits paid was 6,774. As a second nurse was not engaged until November, most of this arduous work devolved upon Miss Wood. The total income of the association in the year amounted to £156 15s., and the total expenditure to £128 10s. 8d., leaving a balance of £28 4s. 4d. in the hands of the treasurer. The speeches at the meeting were marked by considerable enthusiasm, hearty tributes being paid to the valuable services of Miss Wood. The Chairman, very wisely, did not show any disposition to be satisfied with the good results so far obtained, but made a strong appeal to the working men of the town and district to rally round the association, dwelling upon the fact that the subscription of only a halfpenny a week would enable a man, his wife, and his family to have the inestimable advantage of the attendance of a trained nurse in the time of sickness.

### WOMEN NURSES IN WARDS FOR INSANE MEN.

At the tenth semi-annual conference of the Massachusetts State Board of Insanity the question of women nurses in wards for insane men was discussed. Dr. Copp, executive officer of the State Board, introduced the subject, and recited some of the advantages which had followed the introduction of women nurses in wards for insane men. He thought, however, that the whole matter must be determined by the cost. The general opinion of the conference was that in most institutions female nurses might be so employed with profit, but that they should as a rule be under women supervisors. The speakers agreed that in the case of the insane who were in the sick wards, and in that of old men, the female nurses had been found desirable.

### A DUBIOUS EXPERIMENT AT SOUTHPORT.

As the Southport and Birkdale District Nursing Society is not affiliated to Queen Victoria's Jubilee Institute, the committee have a perfect right, if the subscribers do not object, to allow their nurses to attend certain patients for the payment of a small fee. In the report for 1903, it is stated that the result of this innovation, which has only been in operation for a few weeks, has been extremely successful and seems likely to be of great service. In the report, however, there is a passage to the effect that it is not desired in any way to compete with or injure the different nursing institutions in the town. But whatever may be the wish of the committee, the fact remains that a society which was founded in order to nurse the sick poor free of cost, and was provided by the public with funds for the purpose, is receiving fees from some of the patients. The experiment may for a time augment the funds of the organisation, but the danger is that if many cases are nursed for fees, the impression of a considerable number of persons will be that it has ceased to be a charity.

### DISMISSALS AT BEVERLEY WORKHOUSE INFIRMARY.

There having been considerable friction at Beverley Workhouse between the master of the Workhouse and the two nurses in the Infirmary, the House Committee decided to recommend the Guardians to call upon the nurses to resign. This decision appears to have been taken without inquiry, and only in the hope that a change of the nursing staff might prove a peaceful solution of the difficulty. The Board, however, adopted the recommendation of the Committee, and the nurses were accordingly asked to resign. They flatly refused, and asked to know the ground of the Board's decision. The reply of the Board was to give a month's notice to them, with an intimation that no charges had been officially made against them, but that the course had been adopted to put an end to the friction which existed. We think that the Guardians, or the majority who determined to let the matter remain where it is, are wanting in courage. If there be friction of any serious kind in the institution under their care, it is their duty to find out who is responsible, and not to dismiss nurses without accusing them of faults or hearing what they have to say.

### SWANSEA HOSPITAL AND PRIVATE NURSING.

The fears that the private nursing department in connection with Swansea General Hospital would not prove remunerative have been justified. The loss on the half-year lately concluded was £43. At a meeting of the committee Miss Dillwyn, who has been a consistent opponent of the private nursing department, proposed that in consequence of this deficit the branch should be given up. The proposal was negatived, but if the work continues to be carried on at a loss it will have to be reconsidered. The maintenance of a private nursing institution in connection with a hospital supported by the contributions of the charitable is not wise, when the fees from patients cannot be made to balance the cost of keeping it up.

### THE OTHER REGISTRATION BILL.

Mr. Claude Hay has introduced into the House of Commons a Bill to provide for the better training and registration of nurses, and for the voluntary registration of private nursing homes. This is, of course, the measure of the Royal British Nurses' Association, but it has no better prospect than the rival scheme of being debated during the present Session.

### The Nursing Outlook.

"From magnanimity, all fear above;
From nobler recompense, above applause,
Which owes to man's short outlook all its charm.'

### HOSPITAL ECONOMICS.

Misleading paragraphs having appeared in some American and English papers, including The Hospital, about a proposed Course on "Hospital Economics" at Bedford College for Women, in Baker Street, Miss Ethel Hurlbatt, the principal of the college, has kindly supplied all information for the benefit of our readers. First and foremost, the Course would not be undertaken at the suggestion of any one society, nor would it be in any way connected with any one nursing institution. We hope this statement will prepare the minds of London matrons to consider carefully how far the Course can be made useful to such of their staff as show capability of profiting by higher education. Nothing is more depressing nowadays than the lack of women of really high intellect and strong character to fill the administrative posts in the nursing world. We believe that the average nurse is a most excellent person, but a year of post-graduated study at Bedford College might raise some above the average.

In 1895 the authorities of Bedford College started a course of scientific instruction in hygiene which extends over a session of nine months, and includes practical work in chemistry, physics, physiology, and bacteriology in the College laboratories, in addition to hygiene proper. Amongst their students have been some nurses, and from those who hold their hygiene certificate there have been appointed factory inspectors, sanitary inspectors, County Council lecturers, and so on. It is necessary for anyone taking the Course to have had a sound education, including some knowledge of mathematics; but a knowledge of Latin is not necessary. In 1902, papers relating to the Columbia College (New York) teaching in hospital economics were sent to Miss Hurlbatt, and two nursing associations forwarded suggestions with regard to the establishment of a similar Course. Our readers will remember that we also suggested in these pages that Bedford College was the proper institution to help forward the higher education of women in this subject. In January 1903 the subject came before the Board of Education of the college, and in the July following circulars were addressed to all nursing associations stating: "We are advised that our teaching in hygiene and allied subjects may be of use to members of the nursing profession, and we believe that the Course may in part meet the needs of nurses seeking higher qualifications. We shall be glad to receive from you any opinion on the subject." Only eleven replies were received, and none of these was very encouraging, quite half the writers having failed to grasp that the Bedford College Course would have to be taken subsequent to the practical experience in hospital and in addition to all hospital teaching, and that it demands a student's full time for the nine months.

We cannot but conclude that the silence and hesitation on the part of most nurse-training schools was due to a misapprehension, and that now the authorities are assured that the Course is quite independent and solely controlled by the college they will once again consider whether they have not some nurses to whom they could recommend this higher education. It appears that the college is willing, should five nurses enter next October, to make two necessary additions to their existing hygiene Course, and supply 15 lectures on Hospital Economics and a Course on Practical Dietetics; also to allow attendance at the Psychology course in the Teachers' Training Department. This would add to the practical training of some energetic and intellectual nurse such information as would fit her to become a teacher of probationers and the manager of a hospital or institution on lines which would surely lead to success. From our own knowledge we can state that the Hygiene Certificate of Bedford College, giving evidence of practical scientific work as well as mere attendance at lectures and demonstrations, is of unique value, and is often a determining factor as regards public appointments.

There will never be more than a few nurses who will be able to spare time and money for an advanced course; the actual college fee is very low—27 guineas—but the nine months' work and living in London needs some small capital. Whether it would be possible for training schools, when they found a nurse at the end of two years who seemed to have administrative ability and teaching capacity to let her off her third year in the wards and let her deliberately train for higher posts by a year at Bedford College, is a question the future must decide. But certainly steps must soon be taken in one direction or another to give an outlet for the intellectual nurse, and to supply superintendents and matrons who hold some sort of equivalent for a "nursing degree." We are indebted to Bedford College for having made possible an "Advanced Course for Qualified Nurses;" it now only remains for nurses to avail themselves of the opportunity.

### Lectures on Ophthalmic Nursing.

By A. S. Cobbledick, M.D., B.S.Lond., Senior Clinical Assistant and late House-Surgeon and Registrar to the Royal Eye Hospital.

### LECTURE XXIX.—FURTHER REMARKS ON SIGHT-TESTING. STRABISMUS.

A large proportion of the cases which come up for treatment at an eye hospital out-patient department, if systematically tested, is found to have some error of refraction.

The requisites for sight-testing are:—

Snellen's distant types.

Spectacle-frame and obturator.

Case of lenses and cylinders.

The patient should be seated at a distance of six metres from the types; if the room does not allow of this, a reverse type may be reflected from a mirror at a distance of three metres.

The type should be well illuminated. After adjusting the spectacle-frame the vision of each eye is taken separately, and a note made of it. Then place a low convex sphere + ·25 D) in the frame; this either diminishes the vision, does not alter it, or improves it. When the vision is rendered worse, do not use convex spheres any further; if the vision is not altered or is improved, continue to place higher convex spheres in front of the eye and note the result—*e.g.*, if the patient can read $\frac{6}{6}$ without a glass and can see it equally well on looking through a + ·75 sphere, we say that there is present + ·75 of *manifest* hypermetropia $\left(\frac{6}{6}, + \cdot 75 \text{ Hm}\right)$.

If the vision is improved it is noted thus—*e.g.*, $\frac{6}{6}$, $\overline{\iota}$ + ·75 Sph = $\frac{6}{5}$.

If a low convex sphere makes the vision worse a low plus cylinder should be tried; if this produces no improvement, *but not until then*, place the lowest concave sphere in the frame. Improvement with a low minus glass does not in a young person point to short-sight—myopia—but rather to spasm of the ciliary muscle.

If the distant vision is poor, and the child reads small print held within a few inches of the eye, suspect myopia, and make a note of the concave glass which gives the best vision.

Before going further, it is well to understand the meaning of hypermetropia and myopia. Without going into optics and a long explanation, it can be most easily represented and understood by the following diagrams:—

Fig. 1 represents a normal eye, and shows in a rough manner how parallel rays of light after entering the eye converge to a point *on* the retina and so produce a distinct image.

Fig. 2 represents the hypermetropic eye. It illustrates that the rays of light come to a focus *behind* the retina, with the result that the image is blurred, *i.e.* the eyeball is short in its antero-posterior diameter.

Fig. 3 shows how in the myopic or short-sighted eye the rays of light come to a focus *in front of* the retina, and again diverging produce an indistinct image on the retina.

With the aid of these diagrams it is easy to understand why a convex glass must be used to correct hypermetropia and a concave one to correct myopia.

Considerable care is required in testing children: they remember the letters in the types and will repeat them if they cannot see them; it is therefore important when testing a *squinting* eye to be quite certain that the child is not seeing with the other eye, and different types should be used for each eye.

After the preliminary testing, all children and young adults should be subjected to a retinoscopy (shadow test) under atropine, in order to determine the exact defect and to ensure accuracy in prescribing glasses. In adults the shadow test may be performed after the use of homatropine, three instillations of a solution of gr. iv. ad ℥j at intervals of 10 minutes. This drug is more rapid in its action than atropine, and its effects pass off in 12 or 18 hours.

*Astigmatism* is largely a corneal defect, and consists in its irregular curvature. For this defect it is necessary in many cases to order a cylinder with or without a sphere.

*Presbyopia.*—Between the ages of 40 and 50 years a change takes place in the elasticity of the lens, which prevents the change in shape of the lens produced by accommodation for

Fig. 1.

Fig. 2.

Fig. 3.

near objects. The result is that small print cannot be read, and there is increasing difficulty in threading needles. To remedy this defect a low convex sphere has to be worn for near work, and this needs increasing in strength by about half a dioptre every five years.

After the removal of a cataract a strong lens has to be worn to take on the function of the removed lens, *e.g.*, + 10 or + 12 D for distance, and about + 15 D for reading.

*Strabismus.*—This may be internal or external, and is most manifest on accommodation for near objects. These cases may be divided into those which are due to errors of refraction and those due to nerve lesion.

In the first class, internal strabismus is much the most common form, and is associated with hypermetropia. External strabismus may also be met with in hypermetropia, but is more common in myopia.

An external strabismus is also not an uncommon result of division of the internal rectus tendon for the case of internal strabismus, especially when glasses have not been worn after the operation.

These cases of strabismus do not complain of double vision (diplopia), as might be expected. As the squinting y e is not used, the result is that the vision of the squinting eye deteriorates from disuse.

Paralysis of ocular muscles from nerve disease is common, but the subject is so much associated with neurology that a brief outline suffices.

All these cases complain of double vision, the relation of the two images to each other varying with the muscle or muscles paralysed; the nerve most commonly affected is the sixth cranial, which supplies the external rectus muscle, so that when the affected eye tries to follow an object beyond a point corresponding with its central axis it fails to do so, and two objects are seen.

These attacks of diplopia may be transient, and an early symptom of tabes dorsalis; if they are associated with the Argyll-Robertson pupil (inaction to light, but reaction to accommodation) and absent knee-jerks the diagnosis is certain. The muscles supplied by the third cranial nerve are also paralysed in tabes, cerebral tumours, and cerebral syphilis.

### Hospital and Private Nursing in Madeira.

BY AN OCCASIONAL CORRESPONDENT.

There are many nurses who try to get work abroad during the winter on the Riviera, at Cairo, and elsewhere. I wonder if any have thought of trying their fortunes in Madeira? Of course, at most places in the Riviera there is an institute to which nurses are attached, relieving them of all anxiety as to work and provision while unemployed. In Madeira, unfortunately, there is no establishment of the kind, and every nurse coming here for the season would come at a risk; but if the season were good, as it was last year, she would find plenty of work, especially if she held the L.O.S. or a massage certificate or both. Though there is always the risk of uncertainty, I suppose there are some who would not mind that, and certainly the place has many attractions for those who like novelty. I had practically never left England before, so that my first impressions were all the more vivid. Our boat arrived early in the morning one spring day, and I hurried on deck to catch a first glimpse of Funchal. It appeared very white with the houses all built square, without gables, rather flat, tiled roofs of various shades of red and brown, and all the windows furnished with outside shutters. The houses were principally clustered in the valley at the foot of the mountains; some, however, were wandering up the hills, gradually thinning as they went, until they ceased about two-thirds of the way up.

### Going Ashore.

I hurried over breakfast and went ashore. There is no pier as we understand one in England, but only a good-sized stone jetty, which is dignified by that name. Consequently, the steamers anchor about half a mile out, and passengers are all taken ashore in rowing boats. The scene on the jetty was novel in the extreme. Crowds of Portuguese women in short skirts, brightly-coloured shawls, and ditto handkerchiefs tied over their heads; men and small boys in loose shirts, coloured trousers (generally blue) and big slouch hats of straw or felt. These were all trying either to sell their wares, to beg a stray copper, or to offer themselves as guides to exploring parties. I would have none of them, but tried to find my way to my destination—no easy matter as I did not know the language. In the town were more novelties. The streets and footpaths are all paved with cobble stones, most difficult and unpleasant to walk upon; almost the only vehicle is a square, covered car on runners like a sledge, curtained all round and drawn by two oxen, who are devoid of all harness but a wooden collar attached to the one central shaft.

### Riding in a Cart.

My first experience of a ride in one of these conveyances was thrilling. The roads, especially on a slope, are laid in a succession of diminutive hills and valleys in order to give the oxen a foothold. The effect upon the occupant of the car is not as bad as might be expected until one begins to go down hill; then one holds on for dear life, for the car tries to run a race with the oxen, and to get in front in the most presumptuous way; the oxen take no manner of notice of it, being, indeed, quite unable to control the thing, with only one shaft and no traces; so they leave the matter entirely to the driver, who always walks by the side, and he by a mighty effort pushes the car back to its place. I afterwards discovered a few wheeled vehicles, each drawn by two rough-shod ponies, but they look like the last remaining specimens of the first edition of waggonette ever made, and they go by the richly-deserved nick-name of "bone-shaker."

### Luxuriant Flowers.

That first day it seemed to me there were flowers everywhere. Women in the streets were selling baskets and nosegays of violets, geraniums, arum lilies, roses, and many other blossoms unfamiliar but equally charming. Over every garden wall were clusters of richly-coloured creepers—orange, purple, and red—rendered the more brilliant by the sunshine, so that it was difficult to remember that the month was still only February, and not June as it seemed. The heat also was trying in English winter garments, but that was easily remedied. The novelties did not end here by any means. Each day brought something fresh; in fact, one seemed to have come to another world altogether, which had no connection with any other except that nearly all articles of British manufacture were obtainable in the shops.

### Strange Shopping.

But shopping is a very different affair here to what it is in England. The assistants never try to press a customer to have an article she does not want; neither do they pretend to have the required article if they have it not; they will inform the customer where the article can be obtained, even constituting themselves as guide in many cases; or, what is better still, they will go over to another shop and borrow its stock, which they bring across and show at their own counter. This saves a good deal of trouble. The buying of shoes is not the formal business which it is in England. The shoes are first examined at the counter, and if one pair requires fitting, then a stool is produced, or a chair pointed out in some remote corner, and the customer herself does the trying on without assistance, and reports the result at the counter. Most things are very expensive. To give a few commonplace examples, Petit Beurre biscuits are about 1s. 4d. per lb., one cake of Vinolia soap is 1s. 4d., and sugar cannot be had for less than 6½d. per lb. Of course, like most places, Madeira has advantages and disadvantages; the latter include ants, which get into everything, and a not too particular cleanliness in the streets. Matters are very primitive; such things as dust-carts and mechanical road-sweepers are unknown, and at any rate it saves a great deal of trouble to throw all one's rubbish in the street. On the other hand, for anyone who is fond of flowers there are plenty; and as to sunsets, we are provided with a fresh edition every day.

Nursing Experiences.

As to my work, it naturally varies in many ways from that in England. There are some things we have to do without altogether, such as cylinders of oxygen, and some forms of anti-toxin serum; but one great advantage is the climate, for patients are able to be carried out into the garden much sooner than in England, and it is wonderful how quickly they pick up. My own work is a mixture of hospital and private nursing, which makes a pleasant change. Attached to a small hospital of eight beds, we are often not busy, and sometimes entirely without patients, so that I can then go out to private cases, if necessary. Sometimes the patient is in a private house, sometimes in a hotel, and at others one is alone in a cottage with the patient and a Portuguese servant. The cases are almost entirely medical, and lie principally among the English residents and visitors, not among the Portuguese population.

Hospital Work.

In hospital the patients spend most of the time out of doors when they are able to get up. They are called at 7.30 A.M., and are in the garden in time for eight o'clock breakfast. They stay out until it is dark, having all their meals at a table under a roof of corrugated iron; this is at the edge of the cliff, so that the patients can sit there and watch all the boats coming and going in a southerly direction. If a shower of rain falls, they are perfectly protected, but in the event of heavy rain they are obliged to come indoors. Of course, in this as in all hospitals, the amount of work varies considerably. Sometimes we are so busy for a day or two that meals and sleep must take care of themselves. Private nursing is unsatisfactory in one way because I always go on the understanding that directly I am wanted in the hospital I must return; so that I can never be sure of carrying a case through to the end. The localities of the cases vary a good deal. Sometimes I am up on one hill, then on to another, always, however, returning to the hospital in the valley. One week I took a drive of an hour and a half in a hot, jolting carro, to a far distant house, to find that a mistake had been made. No nurse was wanted but only a cook, so I had to come all the way back again, feeling at the end considerably battered about. But "variety is charming," and on the whole I have no cause to complain.

### Maternity Work in South Africa: Nursing in a Dutch Family.

A nurse in South Africa sends us the following experience of maternity work: To reach my destination I had to come to Kaffir River, and then drive for two hours across the veld. When I arrived at the station, a tall, fair man came towards me. "Are you the nurse I expected?" said he, towering majestically above me; then, growing about ten inches larger round the chest, he added impressively, "I am the magistrate!" At first I thought he was going to run me in, for treason at least, but seeing no one else who seemed to be expecting me, I came to the conclusion that I must be the person he was seeking, and that the sister must have omitted to mention to me his high calling. So we started off across the veld, having first driven through the river, which was so high that it came into the bottom of the cart, which was very messy. There were a lot of little yellow flowers in bloom. The magistrate pointed them out to me, and I said: "They grow rather like vetch, only the leaves look juicier." "They have a very strong scent," said he. "Do they smell sweetly?" said I. "No," he answered vaguely, "no, only wild." I nearly asked "what wildness smelt like?" but refrained. He surveyed me critically for some time, and then said: "When I went to the sister to engage a nurse I told her particularly that I did not wish to have one who would make an exhibition of herself. I have known several nurses, and for a person in my position it would be an exceedingly unpleasant thing to happen, so I made an express agreement with the sister that she should not send me a nurse who would make an exhibition of herself." I made no reply, for viewing myself impartially in a particularly unbecoming uniform, a face bathed in perspiration and freckles, sprinkled lavishly with smuts, I could not help wondering what line my exhibition would be likely to take. At last we reached the Residency, a six-roomed house, furnished sparely, as is the very wise custom out here. We went in to see the patient, and after having said "How do you do" to her the magistrate said: "My dear, have you left the brush and comb and tooth-brush in the spare room ready for the nurse?" "My dear" looked at me doubtfully and then said "Yes," but she thought perhaps the nurse had brought her own, and would prefer those. "Oh, have you got some of your own," he said; "do you prefer those?—well, just as you like." I inspected the articles in question on reaching the room, and wondered who had last used them, for the tooth-brush especially exhibited traces of considerable wear and tear. Generally out here we have early dinner and supper, because the black servants object to staying long after tea, and they very seldom sleep in the house, but usually at the Location, and come down at about six in the morning. But at the Residency we have dinner at seven, which is any time it happens to be ready between six and eight, and is invariably served up without any plates, which seem always to escape the memory of the person who lays the cloth. Last night the dinner consisted of sardines, boiled eggs and tea. The baby cries very much at night, so at last after the third or fourth time that the mother had been kept awake all night, I suggested that I should take it into my room. So this was done, and after we had been in bed about an hour or so the infant started screaming and would not be appeased. I was sitting up in bed, and had been trying to comfort it for some time, when I suddenly heard a slight sound behind me, and looking round saw the magistrate. I do not know at all how long he had been standing there, for he had come in through the door leading into the hall instead of the one that led from the bedroom. I expect I looked rather amazed, for he said a little uncomfortably, "I just came in to see what the baby was crying for." "Well," I said blandly, "it has had a dose of castor oil, and has got the stomach-ache." The patient is very nervous and hysterical, and has a book called "Advice to Mothers." She has developed at the rate of two or three a day, all the symptoms of all the complications mentioned in the book, though I have only been here a week. If the baby cries, she is sure it is going to have convulsions, and will I give it a hot bath at once and am I sure that the little red mark where it has scratched its eye is not the beginning of thrush? She has, I fear, come to the conclusion that English nurses are very hard and unsympathetic, but I consider in this I have done a service to the nursing profession, for I pity anyone who comes to nurse her. But let this not be taken as typical of one's experience in the nursing of Dutch people, for sometimes they are very nice; I have just come from a case where every one was pleasant and considerate.

### Everybody's Opinion.

[Correspondence on all subjects is invited, but we cannot in any way be responsible for the opinions expressed by our correspondents. No communication can be entertained if the name and address of the correspondent are not given as a guarantee of good faith, but not necessarily for publication. All correspondents should write on one side of the paper only.]

### THE APPOINTMENT OF A SUPERINTENDENT NURSE.

Miss L. E. Rogers, Superintendent Nurse, The Infirmary, Christchurch, writes: Allow me to contradict the statement made in The Hospital of February 13th that I was trained in an infirmary where there was no resident physician. I was trained in a large training school of 1,400 beds, where there were two resident physicians.

### ALLEGED UNJUST TREATMENT.

"M. R." writes: At a certain county asylum a few weeks ago a charge nurse was publicly insulted by a junior nurse who has only recently joined the staff. The charge nurse reported her complaints to the matron, and she utterly refused to believe her statement, and requested the charge nurse to seek another post. She accordingly applied to the medical superintendent for a testimonial, to enable her to do so, which was as follows: "Nurse —— has been on the staff of this institution since November, 1897, during which time her general character has been good." No mention was made whatever of the way in which she had performed her duties, although for the past six years she had the entire charge of one of the most responsible wards. She can hardly expect to better her position with such a meagre testimonial, and I submit that she has been unjustly treated.

### QUEEN'S NURSES.

"Mrs. E. Nicholson," of Yew Cottage, Egremont, Cumberland, writes: I should be glad if you will kindly allow me to express, through the medium of your paper, my gratitude and admiration of the Queen Victoria's Jubilee Institute for Nurses. I had often heard of the institution and the boon it was to the general public, but never met a "Queen's nurse" until five weeks ago, when my daughter, who was visiting in Appleby, Westmorland, was taken dangerously ill. Then, although I was sent for, as I was a long distance away I could not reach her until the following day, and my anxiety may be imagined. It was in my daughter's sick-room I had the pleasure of meeting the Queen's nurse, and I cannot speak too highly of the splendid nursing she received, moreover, in the nurse we both found a most kind and sympathetic friend, unwearying in her efforts for the patient's comfort and tranquillity of mind. Now we have returned to our home in West Cumberland one of my first duties will be to use my influence amongst my numerous friends to raise subscriptions to support a nurse, for if all the Queen's nurses are like the one at Appleby the sooner we have one the better.

### ASSISTANT NURSES IN WORKHOUSE INFIRMARIES.

"M. C." writes: I was much encouraged by the way in which a "Poor-law Matron" wrote about nurses with a year's training. I also fully understand "Assistant Nurse's" views of the matter. Some time ago, on it becoming vacant, I applied for promotion to one of the higher posts, but the committee disqualified me owing to my certificate, although it had been proved by a somewhat severe test that I possessed the necessary organising abilities. We know in every sphere that there are the incompetent workers, and also that now there is the standard of training, but certainly too much attention is given to the certificate, and the experience and capabilities of the nurse are put on one side. No doubt there is good material out of the number training for assistant nurses, but their certificate will prevent them from rising; consequently their profession will not get the best out of them.

### SIR WILLIAM BENNETT ON PRIVATE NURSING.

"A Dublin Nurse" writes: With reference to the letter of "A Matron," in your columns of 20th inst., this lady, in the course of her remarks, deprecates "unnecessary discussion, and also unseemly discussion," but as she herself has now joined in the correspondence it is evident that she does not include writing upon this subject as coming under either category. Alluding to Sir William Bennett's plain statement, in his address, that "there is no such thing as decency or indecency in the relation of nurse to patient," I think most nurses will agree with the very sensible remarks on the subject of "A Frequent Contributor" in your issue of the 6th inst., but surely it is a very unnecessary reference by "A Matron" to the "lower order of institutions where the matron is untrained," as if the quality of propriety or decency were the property only of a "fully-trained matron," instead of being, as it is, a part of our common womanhood, needing no guidance or training beyond our own sense of what is right and what is wrong. "Cleanliness is next to Godliness," but it is not the cleanliness of soap and water, rather that of thought, word and deed.

"Cantab" writes: With reference to the correspondence "Sir William Bennett on Private Nursing," does it not seem impossible that any person of the gentler sex undertaking nursing as a profession and loving her work could have any scruples as to propriety when taking a male patient? It seems to me that according to the nature of the case if you did to your utmost what was beneficial for your patient, you would not have cause to blush. Certainly if a nurse should so lower herself as to entertain immoral thoughts when her patients were necessarily exposed in person by her she would have herself to blame. As an enteric patient in the Cambridge Military Hospital I had as nurse a civilian lady. To that lady I undoubtedly owed my life. I think that she most certainly did not go beyond the bounds of propriety. I am of opinion that since she determined to aid me with all her power to fight against death, all her actions were such as one expects from true woman. God has specially adapted woman for the loving profession of nursing, and in doing her best for her patients she will never be charged by a right-minded person with immodesty.

"A Frequent Contributor" writes: May I say in answer to "Medico" that the reason nurses "take no account of necessary exposure in the operating theatre" is because the patient is under an anæsthetic, and therefore unconscious. If "Medico" will refer to my letter of February 6th he will see that I wrote "if the patient is conscious a nurse's presence is neither desired nor admitted during the dressing of screen cases." The personal relation of nurse to patient ceases when the patient is no longer a conscious entity. "Medico" argues "if the patient has no objection . . . the nurse should have none either." Is he prepared to follow his argument to its logical conclusion, that the only limit to the extent of a nurse's services to her patient should be fixed by the patient himself? Does he really think that the sister of a ward, who is usually a refined and educated gentlewoman, is not a better judge of the fitness of things than her patients, who in many cases come from homes where overcrowding makes privacy and decency impossible. Again "Medico" says "It is the patient's business to complain, not the nurse's." Why? What decent-minded man would insult a nurse by complaining that she was exceeding the limits of decency? It is for the nurse to stop before those limits are reached. In reply to "Matron's" inquiry I have done no private nursing. I am also at a disadvantage in not having come across nurses "who regale their friends, and anyone else who will listen to them, . . . with ghastly accounts of operations" and whose other unpleasant characteristics "Matron" details. I quite agree with her obvious platitude that these young women "have missed their vocation." I should think their talents would have more scope if applied to the writing of advertisements for quack medicines, or to supplying pseudo-scientific correspondence for the halfpenny press. For "one does not do these things" as a nurse.

### HEALTH VISITORS

"A Health Visitor to a City Council" writes: As a subscriber to your journal for some years, I was pleased to read your article on "Health Visitors." I am one of the visitors in this city, and have been for nearly three years. I tell the people I am glad to be called such, for the health of our people is the first great object, and inspection then follows. What a vast ground for work we have! In some cases we find little improvement; others, after the first few visits, we can lead a step higher and show them why we advocate these hygienic principles, and in this we have been greatly cheered, for there are many that look to us for advice. Only last week I came across a little one of three years no more forward than a baby of eight months. I noticed that the child's spine was weak, and so I told the mother to take her at once to the Orthopædic Hospital and say that I had sent her, without a note. The mother was most grateful to me, as the doctor said it was very serious, and the little one would have to be in a case for a long time. "But Miss," she said, "how was I to know it was so serious, no one ever told me so before?" After the qualification, the success of a visitor depends upon her personality, or tact to apply this knowledge, for there is no mechanical work in our vocation. Each case calls for its own particular treatise. First, you go because you are sent by those who have seen the need of such a work, and with this you have, as it were, a certain amount of authority. Secondly, you go because you have the welfare of the people at heart, and you long to advise them for good and impart some of the knowledge and experience in which you believe; if they follow, they and their offspring must benefit. In your article stress was laid on capturing our future citizens in infancy. I am glad to say that, besides our ordinary work, we now visit all new-born babes in our district, and, to my mind, it is one of the best outcomes of our department. It is most interesting, and so far has been greatly appreciated, for we find it is often for want of knowledge that they feed their babes as miniature men and women, or because "Mother Gamp," or Mrs. So-and-so advised them to do so. I am longing for milk depôts to become universal, and wish that every municipality would see their way to open them in many parts of the town, where the mothers could get milk of known quality for their children cheap. We often find four children under school age with not sufficient money coming in to purchase milk for one. No wonder that there are so many puny, rickety, and malformed. I hope that many able women will go in for this kind of useful work, they will find plenty of scope for their labours. It is a work that is being more and more valued and appreciated here, and the people say that it is one of the best things the Council ever did, and only wonder why it was not started before.

### Presentations.

Buntingford District Nursing Association.—Miss M. B. Hawkes, Queen's Nurse at Buntingford, has been presented on her resignation, after six and a half years' work, with a handsomely fitted travelling bag and a purse containing over £14 from old friends and patients.

### Where to Go.

Midwives Institute, 12 Buckingham Street, Strand.—Lecture by Dr. Mary Rocke to certificated midmives, on Tuesdays, March 8, 15, and 22, at seven o'clock.

### TRAVEL NOTES AND QUERIES.

By Our Travel Correspondent.

Wensleydale in July (M. H. D.).—I am always pleased to hear of the success of my recommendations. It is unfortunately not very easy to find anything so cheap in England to be equally good. I do know Wensleydale, and stayed there a few years since in a small farm house, where I was extremely comfortable. I cannot remember the name of the hamlet, but I think I can have all particulars recalled to my mind by the friend who was with me, and whose memory is not obscured by too much business. As you have plenty of time, watch this column and I will get the information for you. It is a rather out of the way locality, and I do not know that I care for it much. The same summer I stayed at Barnard Castle and greatly preferred it; it is much more convenient for excursions and the surrounding country delightful. The woods are so charming and surround the little old world town Write me again what you wish to pay.

### Appointments.

[No charge is made for announcements under this head, and we are always glad to receive, and publish, appointments. The information to insure accuracy should be sent from the nurses themselves, and we cannot undertake to correct official announcements which may happen to be inaccurate. It is essential that in all cases the school of training should be given.]

Birmingham and Midland Eye Hospital.—Miss Jessie Elms has been appointed sister. She was trained at the Hahnemann Hospital, Liverpool, and has since been charge nurse at the Liverpool Eye and Ear Hospital, and sister at the Central London Ophthalmic Hospital.

Bradford Children's Hospital.—Miss Florence Gower has been appointed sister. She was trained at Salford Royal Hospital and has since been staff nurse at the Children's Hospital, Pendlebury, Manchester, and temporary sister at the Victoria Children's Hospital, Hull.

Hartley Wintney Union Infirmary, Winchfield.—Miss Mary Sansum has been appointed assistant nurse. She was trained at St. Peter's Home, Maybury Hill, Woking, by the Meath Workhouse Nursing Association.

Morpeth Cottage Hospital.—Miss Marjorie Cotter has been appointed matron. She was trained at Fulham Infirmary, and has since been charge nurse at a nursing home in Notting Hill, London, and charge nurse at Bush Fever Hospital, Woolwich. She has also done private nursing.

Western Infirmary of Glasgow.—Miss Ruth Marsh has been appointed superintendent of night nurses. She was trained at Leeds General Infirmary, and has since been sister and night superintendent at Chester General Infirmary.

### The Nurses' Bookshelf.

Garden Cities of To-morrow. Being the Second Edition of "To-morrow." By E. Howard. (Sonnenschein and Co. 1s. net.)

Mr. Howard, in his thoughtful and well-expressed chapters on the solution of the important question, how to stop the migration of the agricultural population into the towns, writes on a subject fraught with grave issues to the community. In place of the universally accepted idea that it is impossible for working men who live in the country to follow pursuits other than agriculture, that if high wages are to be earned they must live in the vicinity of industrial centres, he offers the attractive alternative "of energetic and active town life, with the beauty and delight of the country," and he looks forward to the certainty of their being able to live this life "as a magnet which will produce the effect for which we are all striving—the spontaneous movement of the people from our crowded cities to the bosom of our kindly mother earth, at once the source of life, of happiness, of wealth, and of power." In the formation of "town-country" colonies, fully described by diagrams and detailed descriptions, Mr. Howard finds the clue to the question. There are to be fields and parks of easy access, social opportunities, low rents, high wages, plenty to do, no sweating, no smoke, no slums, bright homes, pure air and water, beautiful gardens, freedom and co-operation. Everyone interested in the subject should read this clearly and cleverly conceived plan for a modern Utopia which will meet the difficulty and arrest the tide of migration townwards. Mr. Howard knows his subject, and treats it in an attractive and practical manner.

### Echoes from the Outside World.

### The Royal Visit to Cambridge.

ON Tuesday the King and Queen visited Cambridge in order to open the buildings in which the schools of geology and botany, of law and medicine, will be housed. Previous visits of reigning sovereigns to Cambridge in State were those of George II., who on the occasion was presented not only with an address, but also with a purse of gold; and of Queen Victoria, who, accompanied by the Prince Consort, drove in 1843 through Tottenham, Waltham Cross, and Royston, to Cambridge, with an escort of yeomanry and of mounted gentlemen, the whole numbering some 5,000. Queen Victoria also visited Cambridge in 1847 when the Prince Consort wa s made Chancellor of the University. The town was generally and gaily decorated on Tuesday, and along the long line of the Royal procession through the streets, brightened by sunshine, to the Senate House, the utmost enthusiasm was manifested. An address of welcome from the Borough Council and the Cambridgeshire County Council was presented at the railway station, and from the Senate. When the latter had been read by the Vice-Chancellor, his Majesty said in his reply that he received with much gratification their renewed expressions of loyalty to his throne and person. He proceeded to declare amid immense cheering, that he was proud of being a member of the University. He looked back with pleasure upon the time he spent as an undergraduate of Trinity, and it was a great satisfaction to him to have sent his dear son, the Duke of Clarence, to be matriculated in the same ancient and splendid foundation. He heartily thanked them for t[illegible] welcome of Queen Alexandra and himself, and he earnestly joined with them in the prayer that his Empire might throughout his reign continue in peace and prosperity. The Vice-Chancellor afterwards read a statement regarding the new schools in Downing Street, which the King formally opened, also unveiling a statue of the late Professor Sedgwick.

### The Princess of Wales and the Cyclist.

LAST week when the Princess of Wales was driving down the Mall towards Marlborough House, her carriage came into violent collision with a bicycle ridden by a youth from Clerkenwell. The Royal carriage was being driven at a very moderate pace close to the kerb on the near side of the roadway, and when the mishap occurred the youth was turning the bend sharply on his wrong side. The coachman had just checked the speed of his horses, and raised his whip as a signal to any driver following that he was about to turn into South Yard. The cyclist, bending low down over the "dropped" handle-bars, had ridden full tilt against one of the horses almost before he was observed. He seized a trace to save himself, but fell to the ground, and one of the carriage wheels passed over his hips and stomach. The Princess immediately stopped the carriage, and displaying great solicitude, asked the Countess of Airlie, who was in attendance, to ascertain the extent of the young fellow's injuries, and see that he received attention. She also signified to the police her desire to be informed of his progress. He has since expressed his gratitude to her Royal Highness for the sympathy shown by her.

### The First Inspector-General.

THE King has approved of the appointment of Field-Marshal the Duke of Connaught and Strathearn to be Inspector-General of the Forces and President of the Selection Board. This appointment will, of course, render it necessary for the Duke to leave Ireland where he has been in command of the Forces for some time and is exceedingly popular. He has also held the command in India and at Aldershot. His Royal Highness joined the Army in 1868.

### The War in the Far East.

A REPORT from Admiral Alexeieff to the Czar was issued in St. Petersburg last Thursday with reference to the Japanese attack on Port Arthur. It states that early on the Wednesday morning the enemy made a fresh attempt to attack the battleship *Retvisan* with several torpedo-boats and to submerge in the channel some large steamers charged with explosives. The *Retvisan*, supported by the shore batteries, opened a fierce fire and destroyed, near the entrance to the channel, two steamers which were bearing down on her. Daylight revealed four steamers destroyed in the channel and eight torpedo-boats fleeing towards the warships awaiting them at sea. The Czar has addressed a message to General Kuropatkin wishing him and the Manchurian army, over which he has command, success in the campaign. Last week, it is officially announced, a protocol was signed at Seoul by which Japan guaranteed the independence and integrity of Korea, and engages to reform the administration with the assistance of her own officials.

### The Importance of Limiting the War.

SPEAKING at Woodbridge last Friday evening Lord Selborne, First Lord of the Admiralty, asked his countrymen in watching the war between Russia and Japan to follow the wise advice he saw coming from a French newspaper in Paris. It said, "What is needed for the English people, as for the French, is to exercise the utmost *sang froid*, the utmost calm, the utmost reserve," and, continued Lord Selborne, "for us, as for France, nothing is of more importance than to limit this war. God grant it may not spread. But it is less likely to spread if every Englishman, the whole public, the whole press, feel their responsibility in this matter."

### The New Governor of New Zealand.

THE King has approved of the appointment of Lord Plunket, K.C.V.O., to be Governor and Commander-in-Chief of the colony of New Zealand, in succession to the Earl of Ranfurly. Like his predecessor, Lord Plunket is an Irishman. His father was the late Archbishop of Dublin, and his uncles are Lord Rathmore, Lord Ardilaun, and Lord Iveagh. He was born in December 1864, and was in the diplomatic service from 1889 to 1894, being attached in succession to the Embassies in Rome and Constantinople. In 1900 he became private secretary to Lord Cadogan, then Lord-Lieutenant of Ireland, now he has held the same office under Lord Dudley. In 1894 the new Governor married Lady Victoria Blackwood, youngest daughter of the late Marquis of Dufferin, and in 1897 he succeeded his father in the peerage.

### The Creator of Commissionaires.

ON Friday last the death of Captain Sir Edward Walter took place at Branksome, Dorsetshire. Sir Edward, who was in his 81st year, was the creator of the Corps of Commissionaires, and at one period the only practical friend the discharged and disabled soldiers possessed. He started the movement in 1859, and for some time he personally conducted his squad of eight to service in Westminster Abbey every Sunday morning. He did not appeal to the public for financial aid until 1864, and then only for the purpose of establishing an "Officers' Endowment Fund." His aim was to make the society self-supporting, and by dint of much perseverance he succeeded. To-day there are nearly 3,000 members. For his great but modest services in a most deserving cause, Captain Walter was made a K.C.B. in 1887. His nephew, Major F. E. Walter, who was long associated with his uncle, is carrying on the valuable work which he originated.

### Notes and Queries.

**FOR REGULATIONS SEE PAGE 277.**

*Hospital Training.*

(200) Will you kindly tell me if a young lady now being trained in a Sydney hospital would be able to enter a London hospital without passing any further examination if she can produce the necessary certificate ?—*A. G. L.*

Yes, the certificates of the best Australian training schools are recognised in England.

*Massage.*

(201) I have been informed that the National Hospital has a class of instruction for massage. I should esteem it a favour if you would let me know which is the said hospital, and also for any particulars concerning it ?—*O. H.*

The National Hospital for the Paralysed and Epileptic, Queen's Square, Bloomsbury, S.W. Apply to the Secretary for particulars.

*Post.*

(202) I have finished my training. Have you any vacancies for nurses to live out, or to assist in medical or surgical work ? I want my evenings free, if only for a time, that is if I can obtain suitable work under those conditions.—*L. M. C.*

Advertise and see advertisements for what you want.

*Sanitary Inspector.*

(203) What would be the most useful certificate in sanitary knowledge for a trained nurse to obtain in order to qualify her for such posts as lecturer, health visitor, etc., under the County Council ? How can she best obtain it ?—*K. H.*

The certificate of the Sanitary Institute, Margaret Street, London, W., is one of the most useful. Apply to the Secretary.

*Wry Neck.*

(204) Will you kindly tell me if it is possible for a child of three to outgrow the deformity of wry neck, or would it be necessary for it to undergo an operation ?—*R. M.*

You can obtain a surgeon's opinion and treatment at the Children's Hospital, Pendlebury Dispensary, Gartside Street, Manchester.

*Enteric.*

(205) Are nurses who nurse enteric fever to blame if they contract the disease, and if so, why are they to blame if they have taken all precautions ?—*A Constant Reader.*

When enteric fever is contracted by a nurse from a patient it is usually due to ignorance or want of care on the part of the latter. Where this is not the case there is no room for blame.

*Brain Trouble.*

(206) Can you kindly tell me of any home where a boy of five years old could be sent ? He is at times very noisy and excitable (brain trouble), and the mother, having young babies and a business to look after, cannot attend to him. She could afford to pay a small amount.—*Nurse Jessie.*

If you suspect brain trouble get the advice of a medical man who would also be able to tell you where it would be best to send the child.

*Nurses' Emigration Society.*

(207) Will you kindly give me any information regarding the Nurses' Emigration Society, as I am anxious to go to South Africa by its aid.—*E. E.*

There is no such society, but nurses, amongst others, are helped to emigrate by the South African Expansion Committee, 47 Victoria Street, Westminster, S.W.

### Important Nursing Textbooks.

"The Nursing Profession : How and where to Train." 2s. net ; 2s. 4d. post free.

"A Handbook for Nurses." By Dr. J. K. Watson. 5s. net ; 5s. 4d. post free.

"Practical Guide to Surgical Bandaging and Dressings." By Wm. Johnson Smith, F.R.C.S. 2s. post free.

"The Nurses' Dictionary of Medical Terms and Nursing Treatment." By Honnor Morten. 2s. post free.

"The Human Body : its Personal Hygiene and Practical Physiology." By B. P. Colton. 5s. post free.

"Art of Feeding the Invalid." (Popular Edition). 1s. 6d. post free.

"On Preparation for Operation in Private Houses. By Stanhope Bishop, F.R.C.S. 6d. post free

### For Reading to the Sick.

LEAVE ALL TO GOD.

LEAVE God to order all thy ways,  
And hope in Him, whate'er betide.  
Thou'lt find Him in the evil days  
Thy all-sufficient strength and guide ;  
Who trusts in God's unchanging love,  
Builds on the rock that nought can move.

*G. Neumarck.*

Open our eyes, thou Sun of life and gladness,  
That we may see that glorious world of Thine !  
It shines for us in vain, while drooping sadness  
Enfolds us here like mist ; come, Power benign,  
Touch our chilled hearts with vernal smile,  
Our wintry course do Thou beguile.  
Nor by the wayside ruins let us mourn,  
Who have th'eternal towers for our appointed bourn.

*J. Keble.*

Be this our rule in action, "not what I will, b[illegible] Thou ;" this, in suffering, "not what I, but [illegible] This shall hallow our hopes, this shall hush [illegible] shall ward off disquiet, this shall still our d[illegible] shall preserve our peace, this shall calm anxi[illegible] soothe our aching hearts, this shall give repose [illegible] ness, this (the deeper our trouble) shall be the deeper [illegible] taste of Everlasting Peace and Rest. For it shall "transfuse our will into His supreme Good Pleasure," and we shall be "the friends of God :" for friends have but one will ; yea, we shall be changed into "one Spirit with" Him, sinking our own bounded will in His, receiving into ourselves His Almighty Will.—*E.B.P.*

By net. THE OF THE D'ARCY

How does our will become sanctified ? By conforming itself unreservedly to that of God. We will all that He wills, and will nothing that He does not will ; we attach our feeble will to that all-powerful will which performs everything. Thus, nothing can ever come to pass against our will ; for nothing can happen save that which God wills, and we find in His good pleasure an inexhaustible source of peace and consolation. *Fénelon.*

Our whole trouble in our lot in this world rises from the disagreement of our mind therewith. Let the mind be brought to the lot, and the whole tumult is instantly hushed ; let it be kept in that disposition and the man shall stand at ease in his affliction, like a rock unmoved with waters beating upon it. *T. Boston.*

The circumstances of her life she could not alter, but she took them to the Lord, and handed them over into His management ; and then she believed that He took it, and she left all the responsibility and the worry and anxiety with Him.—*H. W.S.*

And yet these days of dreariness are sent us from above ;  
They do not come in anger, but in faithfulness and love ;  
They come to teach us lessons which bright ones could not  
yield,  
And to leave us blest and thankful when their purpose is  
fulfilled. *Anon.*

## Figures and Tables

**Fig. 1. f1:**
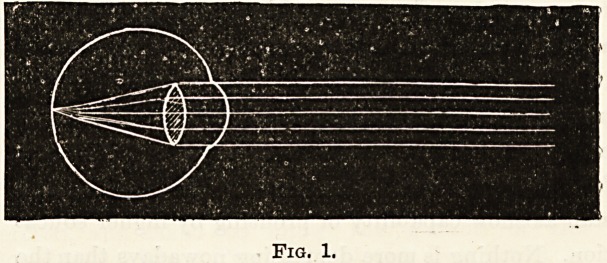


**Fig. 2. f2:**
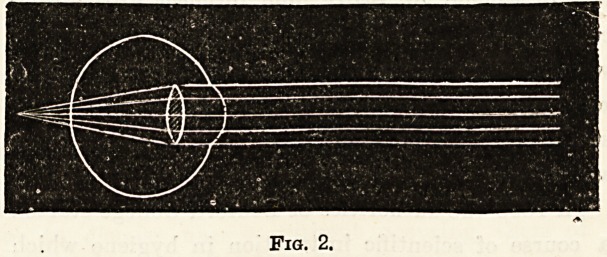


**Fig. 3. f3:**